# Supplementary material for: Reasons for Migration, Post-Migration Sociocultural Characteristics, and Parenting Styles of Chinese American Immigrant Families
Source: Children (Basel). 2023 Mar 24;10(4):612. doi: 10.3390/children10040612 (PMC10136785; doi:10.3390/children10040612)
Supplement: Supplementary file 1 [file children-10-00612-s001.zip › children-2244769-supplementary.pdf]

Supplementary Materials

Questionnaire Items for Demographics, Reasons for Migration, Cultural Orientations, and Parenting Styles

Family Demographics and Migration History Questionnaire

1 If you were asked to identify your ethnicity, would you describe yourself as Chinese or Chinese American?

1--- Yes 2 --- No

2 Before we begin, I'd like to ask a few questions to make sure my information about your family is correct so far. What is your current marital status?

1 ---- Never married and not living with a partner
2 ---- Living with a partner but not legally married
3 ---- Married and living together
4 ---4--- Married but not living together
5 ---5--- Divorced
6 ---6--- Widowed

3 What is your relationship to [child's name]? _________

1 ---- Natural (Biological Mother)
2 ---- Adopted Mother
3 ---- Stepmother
4 ---4--- Grandmother
5 ---5--- Other female relative

4 Is your current husband/partner the biological father of [child's name]?

1--- Yes 2 --- No

5 Now I'd like to ask you about [child's name]. What is [child's name]'s birth date?
_______(year) _______ (month) __________ (day)

6 In what country was [child's name] born? ________
[Skip to Q.10 if the answer is "1"]

1 ---- United States
2 ---- Mainland China
3 ---- Hong Kong
4 ---- Taiwan
5 ---- Some other country (Please specify: ________________)

7 How old was [child's name] when (he/she) first came to live in the U.S.? ________

8 Did [child's name] ever go to school in ____ [answer to Q.6]?

1--- Yes 2 --- No

9 How many years of school (kindergarten and above) did [child's name] attend in [answer to Q.6]?
____

10 Next I'd like to ask whether any other children besides [child's name] live in the household?

Child's Name	Child Age

11 How many other adults besides yourself currently live here and have no other home?

First name of adult family member	Relationship with the child

12 How many bedrooms do you have in your home? ___________

13 How long have you lived in this home? ______________

14 Now let's focus on you for awhile. What is your birth date? ____ (Day) ____ (Month) _________ (Year)

15 In what country were you born? _____________ [Skip to Q.22 if answer is "1"]

1 ---- United States
2 ---- Mainland China
3 ---- Hong Kong
4 ---- Taiwan
5 ---- Some other country (Please specify: ______________)

16 How many years have you lived in the US? ___________

17 Look at this list of reasons why some people move to the U.S. Which of these are the reasons why you first came to the U.S? Now I will read each item one at a time. **Just answer yes or no for each reason**. Please tell me if there were there other reasons why you came to the US.

____1. To join family members
____2 . To leave political problems
____3. To leave personal problems
____4. To find a good job or earn a better income
____5. Because your family brought you
____6. To get an education for yourself

____7. To get married
____8. To provide your children with an education or better opportunities
____9. Other reason (Please specify: ______________________________)

18 In the past 3 years, how many times have you gone to [answer to Q.15] for less than 3 months such as for summer vacation, Christmas vacation, a business trip, or to visit family or friends? [SKIP to Q. 20 if "NONE"]

19 How often did you take [child's name] with you on these visits? ___________

20 In the past 3 years, how many times have you traveled to [answer to Q.15] for 3 months or more? This could be to live there for a while, to help a sick relative, or to work on a job. [SKIP TO Q. 22 IF "NONE"] ___________________

21 How many times did you take [child's name] with you on these visits? _______________

22 In what country did you complete your highest level of education? _____________
1 ---- United States
2 ---- Mainland China
3 ---- Hong Kong
4 ---- Taiwan
5 ---- Some other country (Please specify: ________________)

23 What is the highest level of education (number of years) you have completed? ___________
/00-10 --- If answer is 0 through 10 years of school
/11 --- Completed part of high school but didn't finish
/11.5 --- GED
/12 --- High school graduate
/13 --- Some college, vocational or technical school
/14 --- Vocational or technical school graduate
/15 --- Associate degree
/16 --- College degree (BS/BA)
/17 --- Some advanced work, but no graduate degree
/18 --- MS/MA/MBA (Master's Degree)
/19 --- Some work toward doctorate or advanced Degree
/20 --- Doctorate or other advanced degree (i.e., MD, JD, DO, DDS, or PH.D.)

24 Next, I would like to ask about your religion. Tell me which of these best describes your current religious affiliation: _______________
1 – Buddhism
2 -- Christianity
3 --- Judaism
4 --- Islam
5 --- No religious affiliation
6 --- Other (Please specify: _______________)

25 Now I'd like to talk with you about your parents. What is your father's ethnic or racial background?
1 --- Asian
2 --- Hispanic
3 --- African-American
4 --- White
5 --- Biracial (Specify ___________________)
6 --- Other ethnicity or race? (Specify ______________)

26 In what country was your dad born? _______________
1 ---- United States
2 ---- Mainland China

3 ---- Hong Kong
4 ---- Taiwan
5 ---- Some other country (Please specify: ________________)

27 What is your mother's ethnic or racial background?

1 = Asian
2 = Hispanic
3 = African-American
4 = White
5 = Biracial (Specify ________________)
6 = Or some other ethnicity or race? (Specify) ________________

28 In what country was your mom born? ________________

1 ---- United States
2 ---- Mainland China
3 ---- Hong Kong
4 ---- Taiwan
5 ---- Some other country (Please specify: ________________)

29 Now, let's talk about [child's father]'s background. What is [child's father]'s ethnic or racial background?

1 --- Asian
2 --- Hispanic
3 --- African-American
4 --- White
5 --- Biracial (Specify ________________)
6 --- Other ethnicity or race? (Specify ________________)

30 In what country was [child's father] born? ________________

1 ---- United States
2 ---- Mainland China
3 ---- Hong Kong
4 ---- Taiwan
5 ---- Some other country (Please specify: ________________)

31 How long has [child's father] lived in the U.S.? ________________

32 In what country did [child's father] complete his highest level of education? ____________

1 --- United States
2 --- Mainland China
3 --- Hong Kong
4 --- Taiwan
5 --- Some other country (Please specify: ____________)

33 What is the highest level of education (number of years) [child's father] completed?

/00-10 ---If answer is 0 through 10 years of school
/11 --- Completes part of high school but doesn't finish
/11.5 --- GED
/12 --- High school graduate
/13 --- Some college, vocational or technical school
/14 --- Vocational or technical school graduate
/15 --- Associate degree
/16 --- College degree (BS/BA)
/17 --- Some advanced work, but no graduate degree
/18 --- MS/MA (Master's Degree)
/19 --- Some work toward doctorate or advanced Degree
/20 --- Doctorate or other advanced degree (i.e., MD, JD, DO, DDS, OR PH.D.)

34 Now I'd like to talk with you about [child's father]'s parents. Tell me which ethnic or racial group best describes the background of [child's father]'s dad?

1 --- Asian
2 --- Hispanic
3 --- African-American
4 --- White

5 --- Biracial (Specify) ___________________

6 --- Some other ethnicity or race? (Specify) ____________________

35 In what country was [child's father]'s dad born? ________________

1 --- United States
2 --- Mainland China
3 --- Hong Kong
4 --- Taiwan
5 --- Some other country (Please specify: _____________)

36 Which group best describes the ethnic or racial background of [child's father]'s mom?

1 --- Asian
2 --- Hispanic
3 --- African-American
4 --- White
5 --- Biracial (Specify ___________________)
6 --- Some other ethnicity or race? (Specify ____________________)

37 In what country was [child's father]'s mom born? ________________

1 --- United States
2 --- Mainland China
3 --- Hong Kong
4 --- Taiwan
5 --- Some other country (Please specify: _____________)

38 Now let's talk about your work-related activities. By work we mean anything you do to earn money. This would include a job, your own business or self-employment, anything like that where you earn money. This includes part-time, full-time or occasional work. What is your current employment status? [SKIP TO Q.40 IF NOT CURRENTLY EMPLOYED]

1 ---- Employed full-time
2 ---- Employed part-time
3 ---- Employed occasional / day labor
4 ---- Unemployed but looking for work
5 ---- Unemployed not looking for work
6 ---- Laid off and not currently looking for work
7 ---- Disabled
8 ---- Full-time student and not employed
9 ---- Homemaker and not employed
10 ---- Retired

____

39 Now I would like to know what you do for work. If you have more than one job, I'd like to know about your primary job. What is your occupation? What are your main tasks and responsibilities?

____

[if not currently employed]

40 Since you are not currently employed, what was your main occupation in the past? What were your main tasks and responsibilities?

____

41 What is [child's father]'s current employment status? [SKIP TO Q.42 IF NOT EMPLOYED]

1 ---- Employed full-time
6 ---- Laid off and not currently looking for work

2 ---- Employed part-time
3 ---- Employed occasional / day labor
4 ---- Unemployed but looking for work
5 ---- Unemployed not looking for work
7 ---- Disabled
8 ---- Full-time student and not employed
9 ---- Homemaker and not employed
10 ---- Retired

42 What is [child's father]'s main occupation? What are his main tasks and responsibilities?

____

[If not currently employed]
43 Since [child's father] is not currently employed, what was his main occupation in the past? What were his main tasks and responsibilities?

____

44 Now, I would like you to estimate your total family income for the past year. Think about your combined family income from all sources, including jobs and self employment for you and other adults who contribute to household expenses, as well as money from other sources like welfare, disability benefits, and child support. What is your best guess of this amount for the past 12 months?

/01… Less than or equal to $5000
/02.... $5000 – 10,000
/03.... $10,001 – 15,000
/04.... $15,001 – 20,000
/05.... $20,001 – 25,000
/06.... $25,001 – 30,000
/07.... $30,001 – 35,000
/08.... $35,001 – 40,000
/09.... $40,001 – 45,000
/10.... $45,001 – 50,000
/11.... $50,001 – 55,000
/12.... $55,001 – 60,000
/13.... $60,001 – 65,000
/14.... $65,001 – 70,000
/15.... $70,001 – 75,000
/16.... $75,001 – 80,000
/17.... $80,001 – 85,000
/18.... $85,001 – 90,000
/19.... $90,001 – 95,000
/20.... $95,001+

45 Is [child's name] currently receiving free or reduced meal at his/her school? _________
1 --- No 2 --- Yes

Finally, I'll be asking you some questions about [child's name]'s language proficiency. In these following questions, "Chinese" refers to any Chinese dialect (e.g., Mandarin, Cantonese, Taiwanese).

46. When [child's name] first started to speak, which language did he/she speak?
1--- Chinese 2--- English 3--- Both Chinese and English

47. What language is mostly spoken in your home?
a. Among/between adults (e.g. father-mother, parents-grandparents)
1--- Chinese 2--- English 3--- Both Chinese and English

b. From adult to [child's name]
mother to [child's name] **1--- Chinese** **2--- English** **3--- Both Chinese and English**

father to [child's name] **1--- Chinese** **2--- English** **3--- Both Chinese and English**
other adult to [child's name] **1--- Chinese** **2--- English** **3--- Both Chinese and English**

c. From [child's name] to adult
[child's name] to mother **1--- Chinese** **2--- English** **3--- Both Chinese and English**
[child's name] to father **1--- Chinese** **2--- English** **3--- Both Chinese and English**
[child's name] to other adult **1--- Chinese** **2--- English** **3--- Both Chinese and English**

d. Among children (SKIP if [child's name] is an only child)
1--- Chinese 2--- English 3--- Both Chinese and English

48. At home, would you say [child's name] speaks:
1--- only Chinese
2--- only English
3--- both Chinese and English but better Chinese
4--- both Chinese and English but better English
5--- Chinese and English equally well

49. How old was [child's name] when he/she was first exposed to English?
Age:_____ Type of Instruction/Exposure: ____

50. How old was [child's name] when he/she was first exposed to Chinese?
Age:_____ Type of Instruction/Exposure: ____

51. Is [child's name] currently receiving any kind of formal instruction in **Chinese** (outside of regular school)?
1--- Yes 2--- No

If yes, what kind? (e.g., tutor, Saturday school, afterschool classes) ___________________________
How many times per week? _____________________
In what area (s)? (speaking ,writing, reading)? _____________________

52. Is [child's name] receiving any **English language** instruction outside of regular schooling?
1--- Yes 2--- No

If yes, what kind? (e.g., tutor, Saturday school, afterschool classes) ___________________________
How many times per week? _____________________
In what area? (speaking ,writing, reading)? ____________________

53. Does [child's name] attend any extracurricular or community activities?
Extracurricular activities (e.g., music/dance/art lessons, sports teams, after school programs)
list each activity and its primary language ____
____

Community activities (e.g., community center, church)
list each activity and its primary language ____
____

54. Do you or another adult in your home read to [child's name]?
1--- Yes 2--- No

If yes, how often?
1--- every day 2--- 2-3 times a week 3--- once a week 4--- 2-3 times a month 5--- once a month

If yes, in what language(s)?
1--- Chinese 2--- English 3--- Both Chinese and English

55. How many children's books do you have in your home **in Chinese?**
1--- 1-10 2--- 11-25 3--- 25 or more

56. How many children's books do you have in your home **in English**?
1--- 1-10 2--- 11-25 3--- 25 or more

57. Do you take [child's name] to the library?
1--- Yes 2--- No

If yes, how often?
1--- every day 2--- 2-3 times a week 3--- once a week 4--- 2-3 times a month 5--- once a month

If yes, do you bring home books in Chinese, English, or both?
1--- Chinese 2--- English 3--- Both Chinese and English

58. What is [child's name]'s favorite television show, DVD, or video that he/she watches most often?
GS: indicate child's name of program, type (e.g. TV/DVD) and its language
____
____

59. What other shows, videos, or DVDs does [child's name] watch often?
GS: indicate child's name of program, type (e.g. TV/DVD) and its language
a.
b.
c.

60. On average, how many hours of **Chinese** language TV/DVDs/videos does [child's name] watch every day? ________

61. On average, how many hours of **English** language TV/DVDs/videos does [child's name] watch every day? _________

62. How important is it for [child's name] to be able to **speak Chinese** and/or **English?**

Speak CHINESE	Speak ENGLISH
☐ 5--very important	☐ 5 -- very important
☐ 4	☐ 4
☐ 3	☐ 3
☐ 2	☐ 2
☐ 1--not important at all	☐ 1-- not important at all

63. How important is it that [child's name] learns to **read and write** in **Chinese** and/or **English**?

Read and write in CHINESE	Read and write in ENGLISH
☐ 5--very important	☐ 5 -- very important
☐ 4	☐ 4

CHINESE skills	ENGLISH skills
☐ 3	☐ 3
☐ 2	☐ 2
☐ 1--not important at all	☐ 1-- not important at all

64. How **satisfied** are you with [child's name]'s level of **Chinese skills / English skills?**

CHINESE skills	ENGLISH skills
☐ 5--very satisfied	☐ 5 -- very satisfied
☐ 4	☐ 4
☐ 3	☐ 3
☐ 2	☐ 2
☐ 1--not satisfied at all	☐ 1-- not satisfied at all

Cultural and Social Acculturation Scale

Please mark an "X" on the number next to the answer that best describes your current situation or opinion:

		1 – None	2 – 1 to 3	3 – 4 to 6	4 – 7 or above
1.	How many Caucasian-American friends do you have?	1	2	3	4
2.	How many Chinese friends do you have?	1	2	3	4

		1 – Almost never	2 – Less than once a month	3 – About once a month	4 – About once a week	5 – More than once a week
3.	How often do you invite your Caucasian-American friends to your house?	1	2	3	4	5
4.	How often are you invited to your Caucasian-American friends' gatherings?	1	2	3	4	5
5.	How often do you invite your Chinese friends to your house?	1	2	3	4	5
6.	How often are you invited to your Chinese friends' gatherings?	1	2	3	4	5

		1 – None	2 – 1 to 2	3 – 3 to 4	4 – 5 to 6	5 – 7 or more
7.	To how many formal or informal American organizations (e.g., health club, golf club, etc.) do you belong?	1	2	3	4	5

		1 – American friends	2 – Chinese friends or relatives	3 – Both of them	4 – Neither of them
8.	When you have a problem, you prefer to talk to your:	1	2	3	4

	**5 – Very much**					
	**4 – Somewhat**					
	**3 – Not sure**					
	**2 – A little**					
	**1 – Not at all**					
9.	Do you enjoy being with Caucasian-Americans?	1	2	3	4	5

	**5 – Very good**					
	**4 – Good**					
	**3 – Average**					
	**2 – Poor**					
	**1 – Extremely poor**					
10.	How well do you speak English?	1	2	3	4	5
11.	How well do you understand spoken English?	1	2	3	4	5
12.	How well do you read in English?	1	2	3	4	5
13.	How well do you write in English?	1	2	3	4	5
14.	How well do you speak in Cantonese or Mandarin?	1	2	3	4	5
15.	How well do you understand spoken Cantonese or Mandarin?	1	2	3	4	5
16.	How well do you read in Chinese?	1	2	3	4	5
17.	How well do you write in Chinese?	1	2	3	4	5

	**6 – Almost everyday**						
	**5 – 4 to 5 times a week**						
	**4 – 2 to 3 times a week**						
	**3 – About once a week**						
	**2 – About once or twice a month**						
	**1 – Almost never**						
18.	How often do you read English newspapers?	1	2	3	4	5	6
19.	How often do you read Chinese newspapers?	1	2	3	4	5	6
20.	How often do you watch English movies {including rental movies)?	1	2	3	4	5	6
21.	How often do you watch Chinese movies {including rental movies)?	1	2	3	4	5	6
22.	How often do you listen to English radio?	1	2	3	4	5	6
23.	How often do you listen to Chinese radio?	1	2	3	4	5	6
24.	How often do you watch English TV?	1	2	3	4	5	6
25.	How often do you watch Chinese TV?	1	2	3	4	5	6
26.	How often do you listen to Western music?	1	2	3	4	5	6

27.	How often do you listen to Chinese music?	1	2	3	4	5	6
28.	How often do you go to a Chinese restaurant?	1	2	3	4	5	6
29.	How often do you go to a Western restaurant?	1	2	3	4	5	6

	**3 – All the time**			↓
	**2 – Sometimes**		↓	
	**1 – Almost never**	↓		
30.	Do you require your child to speak Cantonese/Mandarin at home?	1	2	3
31.	Do you celebrate Chinese festivals (e.g., Chinese New Year, Mid-Autumn Festival, etc.)?	1	2	3
32.	Do you celebrate Western festivals (e.g., Christmas, Thanksgiving, etc.)?	1	2	3

Please mark an "X" on the number next to the answer that best describes your child's current situation:

					**4 – 7 or above**
				**3 – 4 to 6**	
			**2 – 1 to 3**		
		**1 – None**			
1.	How many Caucasian-American friends does your child have?	1	2	3	4
2.	How many Chinese friends does your child have?	1	2	3	4

						**5 – More than once a week**
					**4 – About once a week**	
				**3 – About once a month**		
			**2 – Less than once a month**			
		**1 – Almost never**				
3.	How often does your child have "Caucasian-American friends over to your house?	1	2	3	4	5
4.	How often is your child invited to his/her Caucasian- American friends' gatherings?	1	2	3	4	5
5.	How often does your child have Chinese friends over to your house?	1	2	3	4	5
6.	How often is your child invited to his/her Chinese friends' gatherings?	1	2	3	4	5

						**5 – 7 or more**
					**4 – 5 to 6**	
				**3 – 3 to 4**		
			**2 – 1 to 2**			
		**1 – None**				
7.	In how many extracurricular activities (e.g., piano, ballet, gymnastics, etc.) that are taught in English does your child participated?	1	2	3	4	5

					**4 – Neither of them**
				**3 – Both of them**	
			**2 – Chinese friends or relatives**		
		**1 – American friends**			
8.	When your child feels upset about something, he/she prefers to talk to his/her:	1	2	3	4

		**5 – Very**

	much					
	4 – Somewhat					
	3 – Not sure					
	2 – A little					
	1 – Not at all					
9.	Does your child enjoy being with Caucasian-Americans?	1	2	3	4	5

	**5 – Very good**					
	**4 – Good**					
	**3 – Average**					
	**2 – Poor**					
	**1 – Extremely poor**					
10.	How well does your child speak in English?	1	2	3	4	5
11.	How well does your child understand spoken English?	1	2	3	4	5
12.	How well does your child read in English?	1	2	3	4	5
13.	How well does your child write in English?	1	2	3	4	5
14.	How well does your child speak in Cantonese/Mandarin?	1	2	3	4	5
15.	How well does your child understand spoken Cantonese/Mandarin?	1	2	3	4	5
16.	How well does your child read in Chinese?	1	2	3	4	5
17.	How well does your child write in Chinese?	1	2	3	4	5

	**6 – Almost everyday**						
	**5 – 4 to 5 times a week**						
	**4 – 2 to 3 times a week**						
	**3 – About once a week**						
	**2 – About once or twice a month**						
	**1 – Almost never**						
18.	How often does your child look at/read English storybooks, children's magazines, etc.?	1	2	3	4	5	6
19.	How often does your child look/read Chinese storybooks, children's magazines, etc.?	1	2	3	4	5	6
20.	How often does your child watch English movies (including rental movies)?	1	2	3	4	5	6
21.	How often does your child watch Chinese movies (including rental movies)?	1	2	3	4	5	6
22.	How often does your child listen to English radio?	1	2	3	4	5	6
23.	How often does your child listen to Chinese radio?	1	2	3	4	5	6
24.	How often does your child watch English TV?	1	2	3	4	5	6
25.	How often does your child watch Chinese TV?	1	2	3	4	5	6
26.	How often does your child listen to Western music?	1	2	3	4	5	6
27.	How often does your child listen to Chinese music?	1	2	3	4	5	6
28.	How often does your child go to a Chinese restaurant?	1	2	3	4	5	6
29.	How often does your child go to a Western restaurant?	1	2	3	4	5	6

	**3 – All the time**
	**2 – Sometimes**
	**1 – Almost never**

30.	Is your child involved in the celebration of Chinese festivals (e.g., Chinese New Year, Mid-Autumn Festival, etc.)?	1	2	3
31.	Is your child involved in the celebration of Western festivals (e.g., Christmas, Thanksgiving, etc.)?	1	2	3

Parenting Styles and Dimensions Questionnaire

	**5 – Always**					
	**4 – Very often**					
	**3 – About half of the time**					
	**2 – Once in awhile**					
	**1 – Never**					
1.	I encourage my child to talk about his/her troubles.	1	2	3	4	5
2.	I punish by taking privileges away from my child with little if any explanation.	1	2	3	4	5
3.	I tell child our expectations regarding behavior before the child engage in an activity.	1	2	3	4	5
4.	I scold and criticize to make my child improve.	1	2	3	4	5
5.	I joke and play with my child.	1	2	3	4	5
6.	I guide my child by punishment more than by reason.	1	2	3	4	5
7.	I allow my child to give input into family rules.	1	2	3	4	5
8.	I yell or shout when my child misbehaves.	1	2	3	4	5
9.	I know the names of my child's friends.	1	2	3	4	5
10.	I punish by putting my child off somewhere alone with little if any explanation.	1	2	3	4	5
11.	I give my child reasons why rules should be obeyed.	1	2	3	4	5
12.	I tell my child what to do.	1	2	3	4	5
13.	I am easy going and relaxed with my child.	1	2	3	4	5
14.	I spank when my child is disobedient.	1	2	3	4	5
15.	I take my child's desires into account before asking the child to do something.	1	2	3	4	5
16.	I explode in anger towards my child.	1	2	3	4	5
17.	I give praise when my child is good.	1	2	3	4	5
18.	When two children are fighting, I discipline children first and ask questions later.	1	2	3	4	5
19.	I help my child to understand the impact of behavior by encouraging my child to talk about the consequences of his/her own actions.	1	2	3	4	5
20.	I scold or criticize when my child's behavior doesn't meet our expectations.	1	2	3	4	5
21.	I show patience with child.	1	2	3	4	5
22.	I grab my child when being disobedient.	1	2	3	4	5
23.	I encourage my child to freely express him/herself even when disagreeing with parents.	1	2	3	4	5
24.	I argue with my child.	1	2	3	4	5
25.	I show sympathy when my child is hurt or frustrated.	1	2	3	4	5
26.	I use threats as punishment with little or no justification.	1	2	3	4	5
27.	I talk it over and reason with my child when he/she misbehaves.	1	2	3	4	5
28.	I demand that my child do things.	1	2	3	4	5
29.	I show respect for my child's opinions by encouraging my child to express them.	1	2	3	4	5

30.	I use physical punishment as a way of disciplining my child.	1	2	3	4	5
31.	I take into account my child's preferences in making plans for the family.	1	2	3	4	5
32.	I disagree with our child.	1	2	3	4	5
33.	I give comfort and understanding when my child is upset.	1	2	3	4	5
	**5 – Always**					
	**4 – Very often**					
	**3 – About half of the time**					
	**2 – Once in awhile**					
	**1 – Never**					
34.	When my child asks why he/she has to conform, I state: because I said so, or I am your parent and I want you to.	1	2	3	4	5
35.	I explain to my child how we feel about his/her good and bad behavior.	1	2	3	4	5
36.	I tell our child that we appreciate what the child tries or accomplishes.	1	2	3	4	5
37.	I explain the consequences of my child's behavior.	1	2	3	4	5
38.	I am aware of problem or concerns about my child in school.	1	2	3	4	5
39.	I channel my child's misbehavior into a more acceptable activity.	1	2	3	4	5
40.	I express affection by hugging, kissing, and holding my child.	1	2	3	4	5
41.	I apologize to our child when making a mistake in parenting.	1	2	3	4	5
42.	I slap my child when he/she misbehaves.	1	2	3	4	5
43.	I appear to be more concerned with own feelings than with child's feelings	1	2	3	4	5
44.	I am responsive to the child's feelings or needs.	1	2	3	4	5
45.	I have warm and intimate times together with child.	1	2	3	4	5
46.	I emphasize the reasons for rules.	1	2	3	4	5
47.	I bring up child's past mistakes when criticizing him/her.	1	2	3	4	5
48.	I tell my child that his/her behavior was dumb or stupid.	1	2	3	4	5
49.	I show impatience with my child.	1	2	3	4	5
50.	I don't like to be bothered by my child.	1	2	3	4	5
51.	I change mood when with my child.	1	2	3	4	5
52.	I act disappointed when my child misbehaves.	1	2	3	4	5
53.	I tell my child that he/she should be ashamed when he/she misbehaves.	1	2	3	4	5
54.	I tell my child that we get embarrassed when he/she does not meet our expectations.	1	2	3	4	5
55.	I tell my child that he/she is not as good as other children.	1	2	3	4	5
56.	If my child hurts my feelings, I stop talking to my child until he/she pleases me again.	1	2	3	4	5
57.	I am less friendly with my child when my child does not see things my way.	1	2	3	4	5
